# Effects of SGLT-2 Inhibitors on Vascular Endothelial Function and Arterial Stiffness in Subjects With Type 2 Diabetes: A Systematic Review and Meta-Analysis of Randomized Controlled Trials

**DOI:** 10.3389/fendo.2022.826604

**Published:** 2022-02-16

**Authors:** Ran Wei, Weihao Wang, Qi Pan, Lixin Guo

**Affiliations:** ^1^ Department of Endocrinology, Beijing Hospital, National Center of Gerontology, Institute of Geriatric Medicine, Chinese Academy of Medical Sciences, Beijing, China; ^2^ Fifth School of Clinical Medicine, Peking University, Beijing, China

**Keywords:** type diabetes, endothelial function, arterial stiffness, PWV, FMD SGLT-2i, meta-analysis

## Abstract

**Objective:**

This systematic review and meta-analysis aimed to evaluate the effects of SGLT-2 inhibitors (SGLT-2i) on endothelial function and arteriosclerosis in diabetic patients.

**Methods:**

Randomized controlled trials (RCTs) were retrieved from PubMed, Embase, Cochrane Library, and Web of Science databases to evaluate the effects of SGLT-2i on endothelial function and atherosclerosis in type 2 diabetic patients.

**Results:**

We selected 9 RCTs and 2 cohort studys involving 868 patients. Of these, six studies provided flow-mediated dilation (FMD) levels before and after the intervention. The pooled analysis showed that SGLT-2i could significantly improve the FMD compared to the control group (SMD: 0.18, 95% CI: 0.02 ~ 0.34, P = 0.03). Three studies provided the change in FMD before and after the intervention. Pooled analysis showed no significant differences in FMD change between the SGLT-2i group and the control group. (MD: 2.1, 95%-CI: -0.11~4.31, P = 0.06). Five studies showed pulse wave velocity (PWV) results. Pooled analysis showed no significant differences in the change in PWV between the SGLT-2i group and the control group (SMD: 0.11, 95%-CI: − 0.15 ~ 0.37, P = 0.4).

**Conclusions:**

The ability of SGLT-2 inhibitors to improve FMD was significant, but there was no significant effect on PWV levels. SGLT-2i was superior to other antidiabetic agents in improving arterial endothelial function.

## Introduction

Type 2 diabetes mellitus (T2DM) is a chronic disease. According to the International Diabetes Federation (IDF)’s latest global diabetes map (9^th^ edition), the global diabetes prevalence in 20-79 year olds in 2021 was estimated to be 10.5% (536.6 million people), rising to 12.2% (783.2 million) in 2045 (1). The rising prevalence of diabetes poses a global economic burden, especially in developing countries (2). Despite the alarming prevalence of T2DM, approximately 193 million people worldwide still lack diagnosis (3). Diabetic patients are at high risk of developing cardiovascular diseases, constituting the leading cause of death and disability (4, 5). Around the world, approximately 32.2% of T2DM patients are affected by cardiovascular disease. At the same time, T2DM exacerbates the progression of atherosclerosis and heart failure (6).

Diabetic patients at risk for cardiovascular disease generally have endothelial dysfunction and hemodynamic changes in the microcirculation (7). Endothelial dysfunction characterized by reduced bioavailability of nitrous oxide (NO) and oxidative stress is the basis of atherosclerosis. Hence, endothelial dysfunction is a risk factor for atherosclerosis and could predict cardiovascular disease (8). Brachial artery flow-mediated dilatation (FMD) can indirectly measure endothelial function and predict cardiovascular disease in high-risk populations (9). In addition to FMD, pulse wave velocity (PWV) is also used as an evaluation index for the clinical improvement of atherosclerosis in patients with diabetes (10). PWV was included in the new index of hypertension risk classification by the European Society of Cardiology in 2013, and it is the gold standard test for non-invasive evaluation of atherosclerosis (11).

Sodium-glucose cotransporter-2 inhibitors (SGLT-2 inhibitors; SGLT-2i) are a new oral antidiabetic drug developed in recent years. Studies have shown that SGLT-2 inhibitors, such as dapagliflozin, empagliflozin, and canagliflozin, can alleviate arterial stiffness to varying degrees. Results of the Declare-TIMI 58 trial (12) showed that dapagliflozin contributed to lower cardiovascular death rates and heart failure hospitalization compared with placebo. The EMPA-REG trial (13) found a significant reduction in cardiovascular mortality, hospitalization for heart failure, and death from any cause in the empagliflozin group compared with the placebo group. The CANVAS trial (14) confirmed that the composite endpoint of death from cardiovascular causes, non-fatal myocardial infarction, or non-fatal stroke was significantly reduced in the canagliflozin group compared with the control group. Thus, different types of SGLT-2 inhibitors have beneficial effects on cardiovascular disease. However, large-scale clinical studies investigating the effects of SGLT-2 inhibitors on arterial endothelial function and arterial stiffness are lacking. Therefore, the purpose of this meta-analysis was to examine the effects of SGLT-2 inhibitors on endothelial function and arteriosclerosis in patients with T2DM.

## Methods

The Cochrane Handbook for Systematic Reviews of Interventions (15) and Preferred Reporting Items for Systematic Reviews and Meta-Analyses statement (16) were referenced and used as guidelines for reporting the results of our meta-analyses.

### Data Sources, Search Strategy, and Selection Criteria

Based on Population, Intervention, Comparator, Outcomes, and Study (PICOS) design framework, we searched PubMed, Embase, the Cochrane Library, and the Web of Science for SGLT-2i clinical trials that improved endothelial function and atherosclerosis. Keywords, truncation symbols, medical subject heading (MeSH) terms, and Boolean operators (AND/OR) were used in the search strategy. The MeSH table retrieval formula was as follows: “Pulse Wave Analysis” [MeSH] OR “Vascular Stiffness” [MeSH], “Antidiabetic Agents” [MeSH]. The keyword search included the following terms: arterial stiffness index [Title/Abstract] OR pulse pressure index [Title/Abstract] OR ambulatory pulse pressure index [Title/Abstract] OR arterial stiffness [Title/Abstract] OR artery stiffness [Title/Abstract] OR Flow-mediated dilatation [Title/Abstract] OR endothelial function [Title/Abstract] OR pulse wave velocity [Title/Abstract] combined with antidiabetic treatment [Title/Abstract] OR dapagliflozin [Title/Abstract] OR tofogliflozin [Title/Abstract] OR luseogliflozin [Title/Abstract] OR ipragliflozin [Title/Abstract] OR canagliflozin [Title/Abstract] OR empagliflozin [Title/Abstract]. The last search was done on August 24, 2021. The study was included in our meta-analysis if 1) the subjects were clinically diagnosed with T2DM, 2) the study design was a randomized controlled trial, 3) the intervention drug was an SGLT-2 inhibitor, and 4) the endpoints included FMD and PWV values. On the contrary, the study was excluded if it was a cross-sectional study or if data for comparison were incomplete or unavailable.

### Data Collection and Quality Assessment

Potential eligible articles were collected based on the inclusive and exclusive criteria above. The following data was acquired from the selected articles: study characteristics and patient characteristics, including the number of patients, sex, age, BMI, HbA1c, fasting plasma glucose (FBG), medication status, duration, and results, especially FMD (the primary outcome of this analysis) and PWV (the secondary outcome of this analysis).

Based on the above inclusion/exclusion criteria, two independent researchers (Ran Wei and Weihao Wang) reviewed the titles and abstracts of each retrieved paper. If there were any uncertainties regarding qualifications, a third researcher would read the full text. A consensus was reached for all the studies.

### Statistical Analysis

As for some studies only provided the mean and standard deviation of the indicators in the two groups, the data were processed based on the recommendations of the Cochrane Handbook:

(I) For studies that provided only the mean and standard deviation of the indicators, the baseline characteristics were assumed to be equivalent. We combined the mean and standard deviation of each index after treatment using the standardized mean difference method (SMD).
(II) For studies that provided mean and standard deviation of changes in indicators, we combined mean and standard deviation of treatment effects using the weighted mean difference method (WMD). We used 95% confidence intervals (CIs) to represent the size of the difference. We monitored heterogeneity across the studies by the I^2^ statistic, a quantitative measure of inconsistency across studies. A P-value < 0.05 was considered statistically significant regardless of the heterogeneity when using a random effects model. All statistical analyses were processed using Review Manager Software (Rev Man version 5.4; Nordic Cochrane Centre, Cochrane Collaboration).

### Assessment of Bias

The levels of bias of the included articles were evaluated with the Cochrane Collaboration´s tool. We assessed the following factors: random sequence generation (selection bias), blinding of participants and personnel (performance bias), allocation concealment (selection bias), blinding of outcome assessment (detection bias), incomplete outcome data (attrition bias), selective reporting (reporting bias) and other biases. Two independent investigators assessed the bias levels for all the included articles.

## Results

### Selection Process

**Figure 1:**
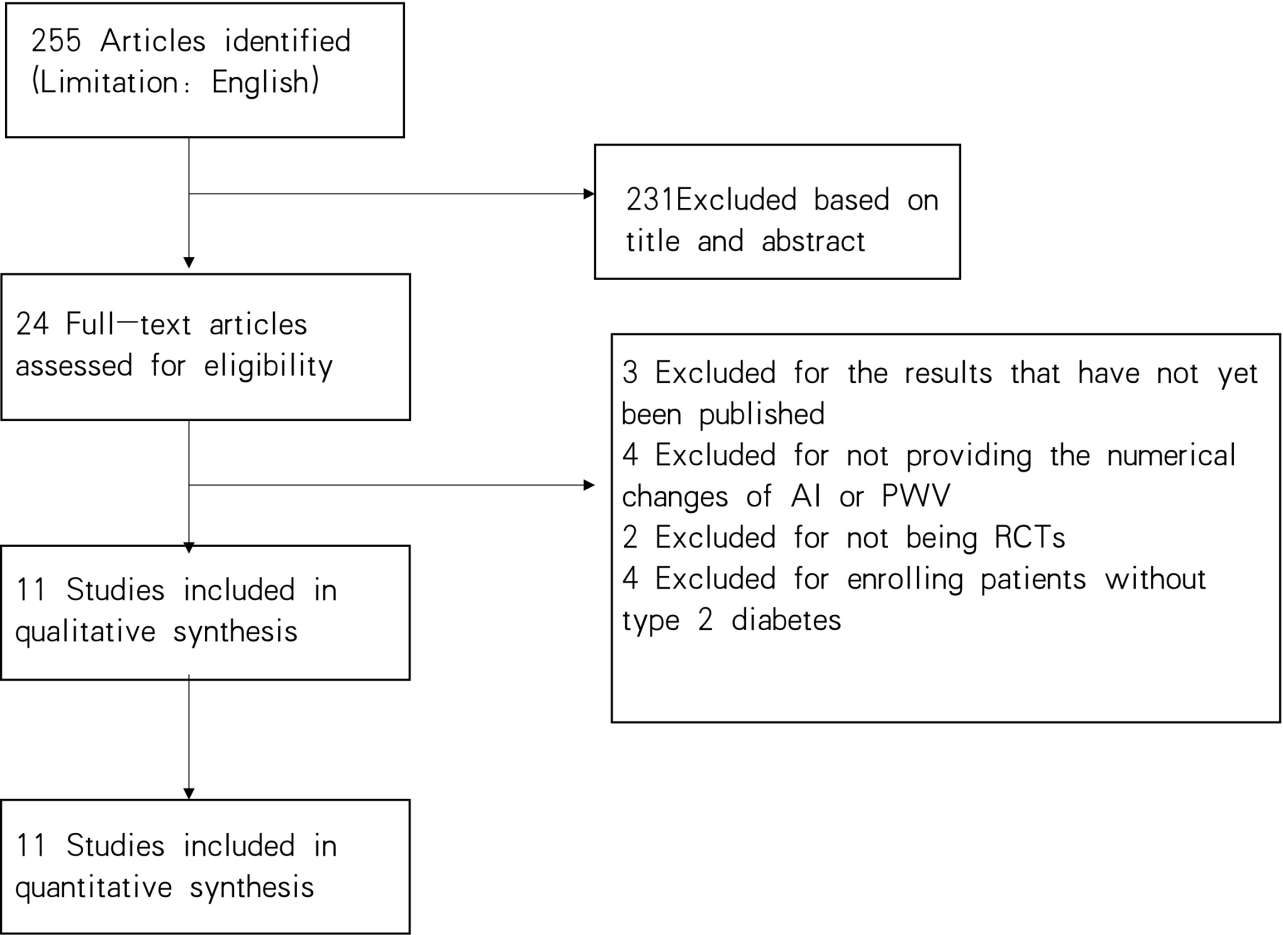
The selection process for the articles included in the meta-analysis.

Two hundred and fifty-five (255) articles were retrieved (159 from Pubmed, 37 from the Cochrane Library, 24 from Web of Science, and 35 from Embase). A total of 24 articles met the inclusive criteria. Of these, 13 were excluded. We excluded three of them because the trials were ongoing, and the results were not published (n = 3) (17–19). Four articles had incomplete data, including PWV and FMD (n = 4) (20–23)and two of them were not randomized or controlled (n = 2) (24, 25). Four trial was excluded because they focused on diseases other than diabetes (n = 4) (26–29). Therefore, 9 RCTs and 2 cohort studys (30–40) were included in our meta-analysis, involving 868 patients and comparing the effects on FMD and PWV with four antidiabetic agents or a placebo. The four antidiabetic drugs were metformin, sulfonylureas, glucagon-like peptide-1 (GLP-1), an incretin. The literature retrieval process is shown in **Figure 1**.

### Characteristics of the Selected Studies

**Figure 2:**
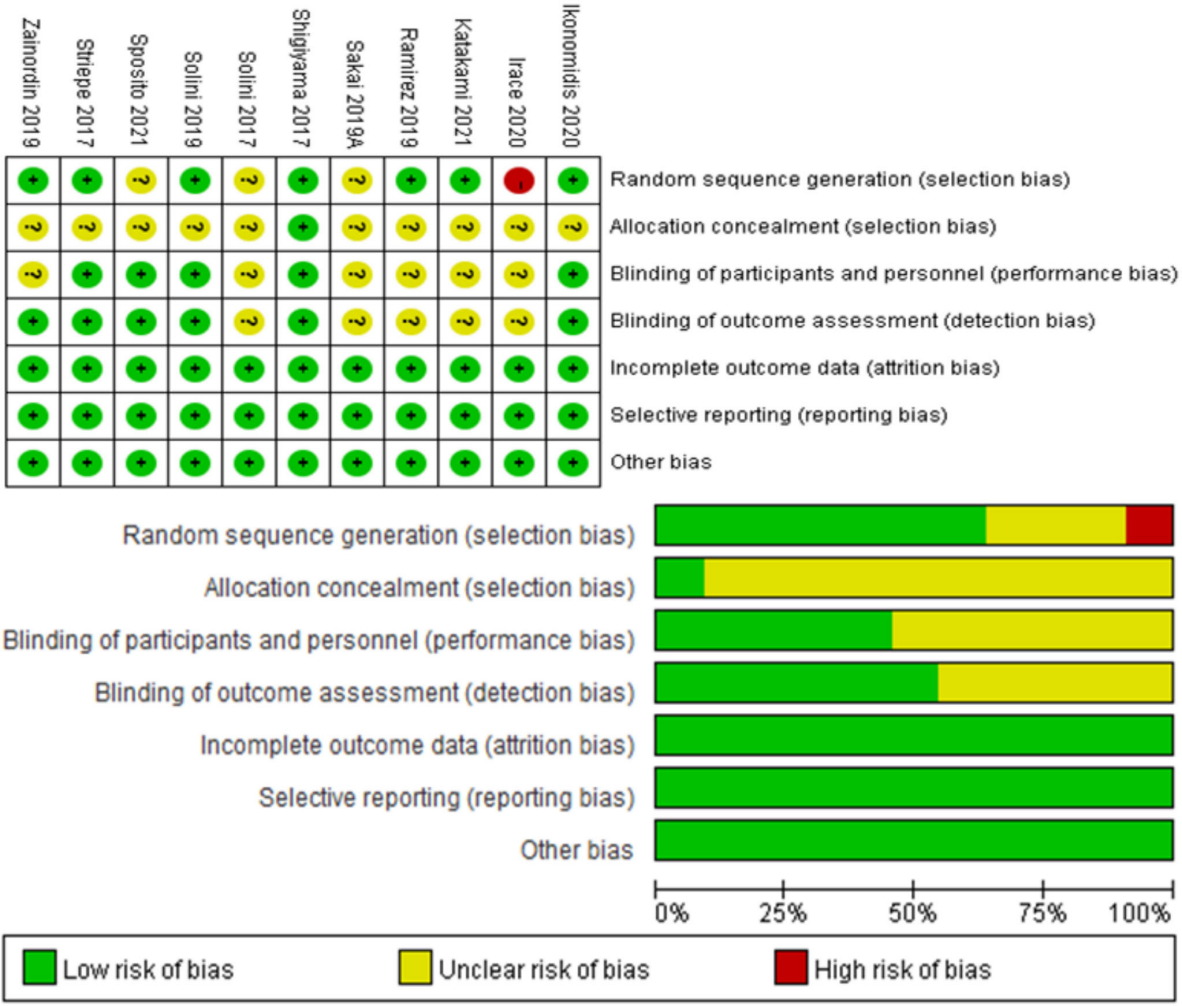
Quality of the studies included in this meta-analysis.

The study characteristics are listed in **Table 1**. This includes first author, year of publication, blood glucose control (including mean HbA1c and FBG), number of patients in the trial, gender of the patients, disease, age, Body Mass Index (BMI), drug name, duration of treatment. The evaluation of the quality of the included studies is presented in **Figure 2**.

**Table 1:** Baseline characteristics of the trials included in the network meta-analysis.

Author	Year	mean HbA1c	FBG(mmol/L)	Number	Male (%)	Age (year)	BMI(kg/m^2^)	Experimental group	Control group	Duration
Sposito (30)	2021	7.9 ± 0.9	9.67 ± 2.4	48,49	29,30	57.0 ± 7.0	31.0 ± 4.0	dapagliflozin	glibenclamide	12 weeks
Zainordin (31)	2019	9.7 ± 1.9	10.69 ± 4.6	36,36	28,27	57.3 ± 8.5	27.5 ± 4.1	dapagliflozin	placebo	12 weeks
Shigiyama (32)	2017	6.8 ± 0.5	7.42 ± 1.5	37,37	25,22	57.9 ± 8.3	26.8 ± 4.6	dapagliflozin	Metformin	16 weeks
Sakai-A (33)	2019	6.8 ± 1.2	8.47 ± 1.7	59,63	36,27	62.0 ± 9.4	27.5 ± 7.9	empagliflozin	luseogliflozin	12 weeks
Sakai-B (33)	2019	6.8 ± 1.2	8.47 ± 1.7	59,62	36,49	62.0 ± 9.4	27.5 ± 7.9	empagliflozin	tofogliflozin	12 weeks
Sakai-C (33)	2019	6.8 ± 1.2	8.47 ± 1.7	63,62	27,49	62.0 ± 9.4	27.5 ± 7.9	luseogliflozin	tofogliflozin	12 weeks
Irace (34)	2020	8.4 ± 0.7	10.30 ± 3.1	20,15	15,12	58.0 ± 9.0	30.0 ± 4.0	empagliflozin	Incretin	3 months
Solini (35)	2017	NA	7.96 ± 2.5	16,10	11,7	57.0 ± 9.0	30.5 ± 6.7	dapagliflozin	hydrochlorothiazide	2 days
Solini (36)	2019	7.5 ± 0.5	8.16 ± 2.0	20,20	12,14	60.0 ± 8.0	32.4 ± 6.7	dapagliflozin	hydrochlorothiazide	4 weeks
Ikonomidis (37)	2020	7.8 ± 0.9	8.06 ± 1.8	40,40	30,27	58.0 ± 10.0	29.8 ± 3.0	SGLT‐2	GLP‐1	12 months
Ramirez (38)	2019	8.1 ± 0.5	8.67 ± 0.6	15,15	8,8	63.0 ± 8.0	NA	Canagliflozin	Perindopril	6 months
Striepe (39)	2017	6.8 ± 0.8	NA	38,38	21,21	62.0 ± 7.0	NA	empagliflozin	placebo	6 weeks
Katakami (40)	2021	7.5 ± 0.7	8.00 ± 1.8	80,74	48,48	61.5 ± 9.2	26.4 ± 5.4	tofogliflozin	conventional	104 weeks

FBG, fasting blood-glucose; NA: not available.

There are **Appendix Table 1 (Supplementary Material)** to interpret the situations of combinative therapy (Angiotensin-converting, Angiotensin II receptor blockers,β-Adrenergic receptor, antiplatelet drugs) of the trials. As shown in **Appendix Table 1 (Supplementary Material)**, four studies (31, 35, 36, 39) did not interpret their concomitant medication, although these drugs may have an impact on the outcomes of FMD and PWV. In five studies (30, 32–34, 37), baseline characteristics of concomitant medication were similar between interventions and control group. Katakami et al. (40) showed that, in the conventional treatment group, more people received angiotensin receptor blockers (P < 0.05). However, mean baPWV was significantly reduced in the tofogliflozin group compared with the conventional treatment group (-104.7 [− 177.0, − 32.4], P = 0.005). Regarding to antiplatelet agents, seven studies (30, 31, 34–36, 38, 39) did not mention the applications of them. In four studies (32, 33, 37, 40), baseline characteristics of antiplatelet agents were similar between interventions and control group.

### The Change in FMD Values After the Treatment

**Figure 3:**
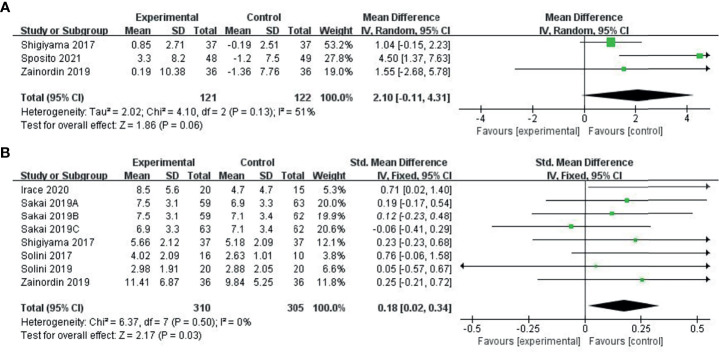
Meta-analysis of articles evaluating the effects of SGLT-2i on FMD levels **(A)** based on the random effects model or **(B)** based on the fixed effects model.

Three studies (30–32) provided changes in FMD before and after drug intervention. We used a random effects model to combine the results. Compared with the control group (glyburide, metformin, and placebo), the ability of the experimental group (SGLT-2i class) to reduce FMD was not significant (MD: 2.1, 950% CI: -0.11-4.31), and we found no statistical significance after combination (P = 0.06), as shown in **Figure 3A**.

Six studies (31–36) provided pre-treatment and post-treatment FMD levels and combined post-treatment PWV values using the SMD method, using a fixed effects model. Compared to the control group (metformin, placebo), the ability of the experimental group (SGLT-2i class) to improve FMD was significant (SMD: 0.18, 95%-CI: 0.02 to 0.34, P=0.03), as shown in **Figure 3B**.

### The Range of PWV Variation Before and After the Treatment

**Figure 4:**
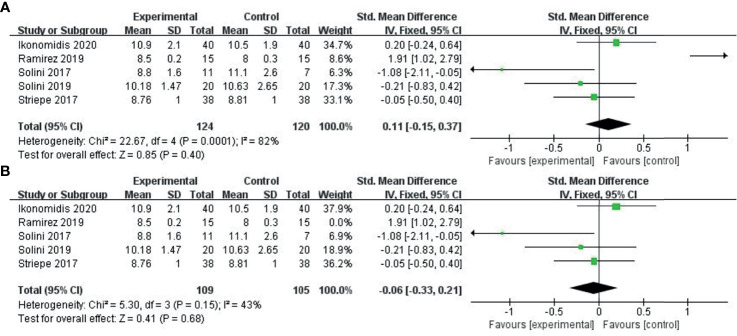
Meta-analysis of articles evaluating the effects of SGLT-2i on PWV levels. **(A)** based on the fixed effects model. **(B)** sensitivity analysis.

Five studies (35–39) provided PWV levels before and after treatment. We combined PWV values after treatment using the SMD method. Due to the small heterogeneity among the studies, we used the fixed effects model. Compared with the control group (GLP-1, perindopril, placebo), the ability of the experimental group (SGLT-2i class) to reduce PWV was not significant (SMD: 0.11, 95%-CI: − 0.15-0.37, P = 0.4), as shown in **Figure 4A**. As shown in **Figure 4B**, the sensitivity analysis results indicated change in the heterogeneity after excluding the study conducted by Ramirez et al. (38), which specifically included patients with diabetes mellitus and hypertension.

## Discussion

The development of atherosclerosis depends mainly on the structure of the tunica media, which contains the elastic components of the aortic wall, namely elastin fibers, and collagen. Arteriosclerosis should be distinguished from atherosclerosis, as the latter is mainly caused by changes in the intima caused by intra-vascular inflammation, lipid oxidation, and plaque formation. Diabetes, hypertension, aging, obesity, inflammation, dyslipidemia, smoking, and hypercholesterolemia are common causes of atherosclerosis. T2DM is a common clinical disease. SGLT-2i is a new oral antidiabetic drug in recent years. Large-scale clinical studies have shown that SGLT-2i has beneficial effects on cardiovascular disease.

The study is a meta-analysis that includes 9 RCTs and 2 cohort studys studies to assess the changes in arterial stiffness in patients with T2DM after treatment with the SGLT-2i class or other types of antidiabetic agents. Two indicators, including FMD and PWV, were used to assess arterial stiffness in the present study. The published literature suggests that research in the area is limited. It gave credit to some studies conducted but not published (17–19). There were few small randomized clinical trials with moderate heterogeneity.

As a consequence, we need to be cautious about their findings. Our results showed that the experimental group (SGLT-2 inhibitor) improved FMD significantly compared with the control group (metformin, placebo) (SMD: 0.18, 95%-CI: 0.02 to 0.34, P = 0.03). Compared with the control group (GLP-1, perindopril, placebo), the ability of the experimental group (SGLT-2 inhibitor) to reduce PWV was not significant (SMD: 0.11, 95%-CI: − 0.15-0.37, P = 0.4). However, many clinical studies have reported that compared to other types of antidiabetic agents, SGLT-2 inhibitors may improve arterial stiffness levels in patients with T2DM. To the best of our knowledge, this is the first meta-analysis to compare the effect of the SGLT-2 inhibitor class of drugs and other types of antidiabetic agents on the improvement of endothelial function and arterial stiffness in patients with T2DM.

The following is an interpretation of our study. First, the ability of the SGLT-2 inhibitor to improve the PWV level was not superior to other types of antidiabetic agents. On the contrary, the ability of the SGLT-2 inhibitor to improve the FMD level was superior to other types of antidiabetic agents. FMD is an indicator of endothelial function, and PWV is an indicator of arterial stiffness. The two factors are entirely different, and endothelial function damage is one of the causes of arteriosclerosis. Therefore, we can further evaluate the effect of SGLT-2 inhibitor drugs on arterial stiffness by investigating the improvement of FMD. Second, from a statistical point of view, as shown in **Figure 4**, the improvement of SGLT-2i in PWV was still more significant than that of other antidiabetic drugs. However, the difference was not statistically significant, mainly due to the small sample size and poor statistical ability, and further investigation is required. Third, the number of studies included is small, and more data is needed to unveil the effects of SGLT-2 inhibitors on arterial stiffness and endothelial function. Whether their impact on vascular function FMD and PWV determine their impact on cardiovascular disease remains to be seen. Fourth, Angiotensin-converting, Angiotensin II receptor blockers,β-Adrenergic receptor, antiplatelet drugs played a vital role in ameliorating vascular endothelial function and arterial stiffness in subjects with type 2 diabetes. As shown in **Appendix Table 1 (Supplementary Material)**, some studies did not interpret their concomitant medication, while other studies’ baseline characteristics of concomitant medication were similar between interventions and control group. Therefore, PWV improvement due to the application of ARB and antiplatelet agents can be excluded. Last but not least, compared with other antidiabetic drugs, SGLT-2i was superior in improving FMD, but not PWV. In general, SGLT-2i was superior to other antidiabetic agents in improving endothelial function.

It is important to note that regardless of the use of hypoglycemic drugs, glycemic variability is a fundamental cause of endothelial dysfunction. This mechanism was confirmed by improved cardiovascular outcomes in acute coronary syndrome patients with strict glycemic control (41, 42). A recent meta-analysis that included 16 studies showed that SGLT-2 inhibitors could effectively reduce the mean amplitude of glucose excursion and glycemic variability (43). Since AT1R/NADPH oxidase/SGLT1 and 2 pathways promote endothelial dysfunction, SGLT-2i provide a promising strategy to ameliorate endothelial function. Other studies of experimental atherosclerosis models have shown that compared to plaques from patients without diabetes, plaques from patients with diabetes had higher SGLT2 expression, inflammation, and oxidative stress, along with lower SIRT6 expression and collagen content. SGLT2/SIRT6 pathway played a critical involvement in the inflammatory process of diabetic atherosclerotic lesions. Therefore, SGLT-2i improves the progression of atherosclerosis by inhibiting vascular inflammation, reducing oxidative stress, reversing endothelial dysfunction, reducing foam cell formation, and preventing platelet activation (44–47). Empagliflozin and dapagliflozin restore the bioavailability of NO by inhibiting reactive oxygen species (ROS) generation, consequently improving endothelial function (48–50). Notably, differences exist between different SGLT2i. Tahara et al. compared the effects of six types of SGLT2i (luseogliflozin, ipragliflozin, tofogliflozin, empagliflozin, canagliflozin, and dapagliflozin) on diabetes-related complications in mice with T2DM. The study determined that all SGLT2i prevented the development of endothelial dysfunction. Two long-acting drugs (dapagliflozin and ipragliflozin) more effectively improved these diabetes-related diseases and complications than four intermediate-acting four drugs (tofogliflozin, canagliflozin, empagliflozin, and luseogliflozin), though without statistically significance (51). Meanwhile, there were too few articles to do a subgroup analysis. Therefore, animal and other basic studies are consistent with the conclusion of our study.

However, our study still has the following limitations: (1) We only retrieved articles published in English, with the possibility of selection bias. (2) We poorly controlled heterogeneity in the experiment. Heterogeneity is substantial in **Figure 4**, due to study conducted by Ramirez et al. (38), which specifically included patients with diabetes mellitus and hypertension. Meanwhile, the PWV data types (ba-PWV or cf-PWV) are different. Meanwhile, in **Table 1**, some data referred to the change of FMD within 1 minute (FMD 1 min), and some data to the maximum change of FMD (peak FMD). Some literature did not explain how the PWV and FMD values were obtained. (3) Some literature was not included in this article due to the lack of PWV and FMD values and incomplete data, resulting in selection bias. (4) The follow-up period for different studies was also different. Previous studies have shown that short-term and long-term drug therapy mechanisms on arterial stiffness differ. Therefore, different follow-up times can affect the study results. Generally speaking, there are still many shortcomings in the study. We urgently need new evidence on the improvement of endothelial function and atherosclerosis in diabetic patients with SGLT-2i.

It is imperative to verify the effect of arterial stiffness by SGLT-2i from the following aspects. Firstly, the changes of endothelial function and arterial stiffness by SGLT-2i with different treatment durations and further analyze whether short-term and long-term antidiabetic drug therapy mechanisms are different. Secondly, it should be further evaluated whether SGLT-2i could affect endothelial function and arterial stiffness independently of changes in blood glucose. Finally, there is a thought-provoking problem of whether SGLT-2i class of drugs may also improve endothelial function and arterial stiffness in T2DM patients with complex clinical conditions such as dialysis, hypertension, and coronary heart disease. Further evaluations of the change of endothelial function and arterial stiffness by SGLT-2i in T2DM patients complicated with other risk factors are essential, especially high-risk patients with cardiovascular disease.

## Conclusions

In conclusion, the present meta-analysis suggests that the quality of the published literature on new antidiabetic drugs and vascular function is moderate, with moderate heterogeneity between studies. Our results showed that the ability of SGLT-2i to improve FMD was significant, and the ability to reduce PWV was not significant compared with the control group due to the poor number of studies included. More data should be acquired on the effects of SGLT-2i on arterial stiffness and endothelial function. SGLT-2i was superior to other antidiabetic agents in improving arterial endothelial function.

## Data Availability Statement

The original contributions presented in the study are included in the article/**Supplementary Material**. Further inquiries can be directed to the corresponding author.

## Author Contributions

RW consulted literature and wrote the manuscript. LG and QP designed the review. WW assisted with writing and revised the manuscript. All authors contributed to the article and approved the submitted version.

## Funding

This work was supported by the National Natural Science Foundation of China (grants 81670763 and 81471050).

## Conflict of Interest

The authors declare that the research was conducted in the absence of any commercial or financial relationships that could be construed as a potential conflict of interest.

## Publisher’s Note

All claims expressed in this article are solely those of the authors and do not necessarily represent those of their affiliated organizations, or those of the publisher, the editors and the reviewers. Any product that may be evaluated in this article, or claim that may be made by its manufacturer, is not guaranteed or endorsed by the publisher.
