# Supplementary material for: Effects of SGLT-2 Inhibitors on Vascular Endothelial Function and Arterial Stiffness in Subjects With Type 2 Diabetes: A Systematic Review and Meta-Analysis of Randomized Controlled Trials
Source: Front Endocrinol (Lausanne). 2022 Feb 16;13:826604. doi: 10.3389/fendo.2022.826604 (PMC8889103; doi:10.3389/fendo.2022.826604)
Supplement: Supplementary file 1 [file Table_1.docx]

| Author | Interventions and  Comparison | statins | Angiotensin-converting enzyme inhibitors | Angiotensin receptor blockers | β-Adrenergic receptor antagonist | Antiplatelet agents |
| --- | --- | --- | --- | --- | --- | --- |
| Sposito  (30) | Dapagliflozin  （%） | 45% | NA | 100% | NA | NA |
|  | Glibenclamide  （%） | 43% | NA | 100% | NA | NA |
| Zainordin  (31) | dapagliflozin | NA | NA | NA | NA | NA |
|  | placebo | NA | NA | NA | NA | NA |
| Shigiyama (32) | dapagliflozin | 14 (37.8) | 4 (10.8) | 6 (16.2) | 1 (2.7) | 1 (2.7) |
|  | Metformin | 15 (40.5) | 0 (0.0) | 11 (29.7) | 0 (0.0) | 1 (2.7) |
|  | P value | 1.00 | 0.11 | 0.27 | 1.00 | 1.00 |
| Sakai (33) | Empagliflozin（%） | 52.5（11.7） | 74.2（16.6） | 74.2（16.6） | 87.4（19.5） | 35.4（7.9） |
|  | Luseogliflozin  （%） | 54.3（12.1） | 65.5（14.6） | 65.5（14.6） | 79.1（17.6） | 31.4（7.0） |
|  | Tofogliflozin  （%） | 47.7（10.7） | 79.1（17.6） | 79.1（17.6） | 88.7（19.8） | 25.3（5.6） |
| Irace  (34) | Empagliflozin  （%） | 100% | 62% | NA | NA | NA |
|  | Incretin  （%） | 100% | 62% | NA | NA | NA |
| Solini  (35) | dapagliflozin | NA | NA | NA | NA | NA |
|  | hydrochlorothiazide | NA | NA | NA | NA | NA |
| Solini  (36) | dapagliflozin | NA | NA | NA | NA | NA |
|  | hydrochlorothiazide | NA | NA | NA | NA | NA |
| Ikonomidis (37) | SGLT‐2 | 40 (100) | 21 (52.5) | 21 (52.5) | 21 (52.5) | 15 (37.5) |
|  | GLP‐1 | 40 (100) | 19 (47.5) | 19 (47.5) | 19 (47.5) | 13 (32.5) |
|  | P value | 1.000 | 0.734 | 0.734 | 0.753 | 0.898 |
| Ramirez (38) | Canagliflozin | NA | 0% | NA | NA | NA |
|  | Perindopril | NA | 100% | NA | NA | NA |
| Striepe  (39) | empagliflozin | NA | NA | NA | NA | NA |
|  | placebo | NA | NA | NA | NA | NA |
| Katakami (40) | tofogliflozin | 33 (41.3) | 1 (1.3) | 25 (31.3) | 3 (3.8) | 10 (12.5) |
|  | conventional | 32 (42.3) | 4 (5.4) | 41 (55.4) | 2 (2.7) | 9 (12.2) |
|  | P value | 0.87 | 0.20 | 0.003 | 1.00 | 1.00 |

Appendix Table 1. Concomitant medication of the trials included in the meta-analysis. NA: not available.
